# Chemical composition and repellent activity of essential oils of *Tithonia diversifolia* (Asteraceae) leaves against the bites of *Anopheles coluzzii*

**DOI:** 10.1038/s41598-023-31791-6

**Published:** 2023-04-12

**Authors:** Cédric Akeumbiwo Tchumkam, Loick Pradel Kojom Foko, Cyrille Ndo, Estelle Essangui Same, Glwadys Cheteug Nguetsa, François Eya’Ane Meva, Lawrence Ayong, Carole Else Eboumbou Moukoko

**Affiliations:** 1 Department of Pharmaceutical Sciences, Inorganic Chemistry Lab, Faculty of Medicine and Pharmaceutical Sciences, The University of Douala, P.O. Box 2701, Douala, Cameroon; 2 Department of Animal Biology, Faculty of Science, The University of Douala, P.O. Box 24157, Douala, Cameroon; 3 Department of Biological Sciences, Parasitology Lab, Faculty of Medicine and Pharmaceutical Sciences, The University of Douala, P.O. Box 2701, Douala, Cameroon; 4 Organisation de Coordination pour la lutte contre les Endémies en Afrique Centrale (OCEAC), Research Institut of Yaoundé, 288 Yaoundé, Cameroon; 5 Malaria Research Unit, Centre Pasteur Cameroon, P.O. Box 1274, Yaoundé, Cameroon; 6 Laboratory of Parasitology, Mycology and Virology, Postgraduate Training Unit for Health Sciences, Postgraduate School for Pure and Applied Sciences, University of Douala, P.O Box 24157, Douala, Cameroon

**Keywords:** Drug discovery, Ecology, Plant sciences

## Abstract

*Tithonia diversifolia* is widely used in African traditional medicine for the treatment of a large number of ailments and disorders, including malaria. In the present study, we evaluated the repellent activity of essential oils (EO) of this plant against *Anopheles coluzzii*, a major vector of malaria in Africa. Fresh leaves of *T. diversifolia* were used to extract EO, which were used to perform repellency assays in the laboratory and in the field using commercially available N,N-Diethyl-meta-toluamide (DEET) and *Cymbopogon (C.) citratus* EO as positive controls and vaseline as negative control. The repellency rates and durations of protection of the human volunteers involved were used as measures of repellent activity. Chemical composition of the *T. diversifolia* EO was established further by gas chromatography coupled with mass spectrometry. The moisture content and oil yield were 81% and 0.02% respectively. A total of 29 compounds in the *T. diversifolia* EO was identified, with d-limonene (20.1%), α-Copaene (10.3%) and o-Cymene (10.0%) as the most represented. In field studies, the mean time of protection against mosquito bites was significantly lower in *T. diversifolia* EO-treated volunteers compared to treatments with *C. citratus* EO (71 min versus 125 min, p = 0.04), but significantly higher when compared with the non-treated volunteers (71 min vs 0.5 min, p = 0.03). The same pattern was found in laboratory repellency assays against *A*. c*oluzzii*. In contrast, repulsion rates were statistically similar between *T. diversifolia* EO and positive controls. In conclusion, the study suggests promising repellent potential of leaves of *T. diversifolia* EO against *A. coluzzii.*

## Introduction

*Anopheles coluzzii* (Diptera: Culicidae) is one of the main mosquito species responsible for malaria transmission in endemic countries^1^. Malaria is an infectious disease caused in humans by protozoan parasites belonging to the genus *Plasmodium*, *Plasmodium falciparum* (*P.f*) and *P. vivax* being the two species accounting for the bulk of malaria burden^2^. The disease is an important public health concern with nearly 229 million cases and 409,000 deaths worldwide, especially in children aged under 5 years and pregnant women^3^. Malaria burden has been dramatically reduced over the past two decades. Between 2000 and 2019, malaria case incidence rates dropped from 362.8 to 225.2 cases per 1000 population at risk. The same pattern was observed for malaria mortality rate which has fallen from 121.1 to 40.3 deaths per 100,000 population at risk during the same period^3^.

These main achievements were the result of implementation and/or scale up of varied malaria control interventions viz. free distribution of long-lasting insecticide treated nets (LLINs), more reliable diagnosis of the infection through immunochromatographic rapid diagnostic tests, and their treatment with artemisinin-based combination therapies (ACTs)^3^.

Unfortunately, malaria burden is still dramatically high in several endemic countries, especially in sub-Saharan Africa which bears > 90% of global morbidity and mortality cases^3^. The emergence and spread of ACT-resistant *P. falciparum* populations and insecticide-resistant *Anopheles* mosquitoes hindered enormous efforts made in malaria control and elimination^4,5^. Additionally, LLIN-based mosquito control in African countries is compromised by the change in biting behavior of *Anopheles* vectors which increasingly choose to bite humans indoors in the early evening or bite outdoor (also known as behavioral resistance), thereby limiting the positive impact of LLINs^6–8^.

Malaria is a cause of concern in Cameroon with a prevalence of 24% in children under five, and accounting for 25.8% of all medical consultations in 2018^9^. Cameroon is also targeted by the WHO “High burden to High impact” strategy which aims at reducing malaria transmission in 11 countries where malaria burden is highest^10^. LLINs are a key control for curbing malaria in the country. The government of Cameroon also encourages the use of additional personal protection measures, including coils, indoor residual sprays, as well as body repellents, all of which are widely used by local populations^11–13^.

Repellents reduce malaria transmission by minimizing mosquito-human contacts. The most commercially available repellent formulations are either synthetic (e.g., N,N-Diethyl-meta-toluamide-DEET) or derived from plant extracts (e.g. Neem, Citronella, fennel or Pyrethrum grasses)^14^. DEET is the oldest and most effective insect repellent available on the market, but several studies have reported occasional mild-to-severe toxicity reactions following the application of this molecule on the skin^15^. Although the above mentioned plant extract-based repellents are less toxic than their DEET-based counterparts, they are less effective^16,17^. Similarly, the effectiveness of *Cymbopogon (C.) citratus*, *Citrus limon*, *Melissa officinalis* essential oils (EO) has been proven in some previous studies, albeit with differences in sensitivity according to vector species^18,19^. In addition, the duration of protection of a repellent varies between individuals depending on the amount of lactic acid excreted in the sweat and other factors of attraction in humans^20,21^. Therefore, more effective and durable repellents are needed to overcome the current challenges facing this key vector control strategy.

In this regard, EOs represent suitable alternatives for repellent development as they are inexpensive, relatively safe and durability under body temperature conditions^22^. In the present study, we evaluated the repellent activity of *T. diversifolia* EOs against *Anopheles coluzzii* mosquitoes. This plant, native to Central America, is now widely distributed in Australia, Asia and Africa^23^. Some studies showed an interesting repellent activity of *T. diversifolia* EO and aqueous extract (AE) against *Anopheles gambiae* s.l.^24,25^, ticks^21^ and against fleas^26^ and were limited to West, East Africa and Asia. To the best of our knowledge, no published study evaluated its repellent activity against *A. coluzzii* that is widely distributed in Africa.

## Methods

### Plant collection and ethnobotanical identification

**Figure 1:**
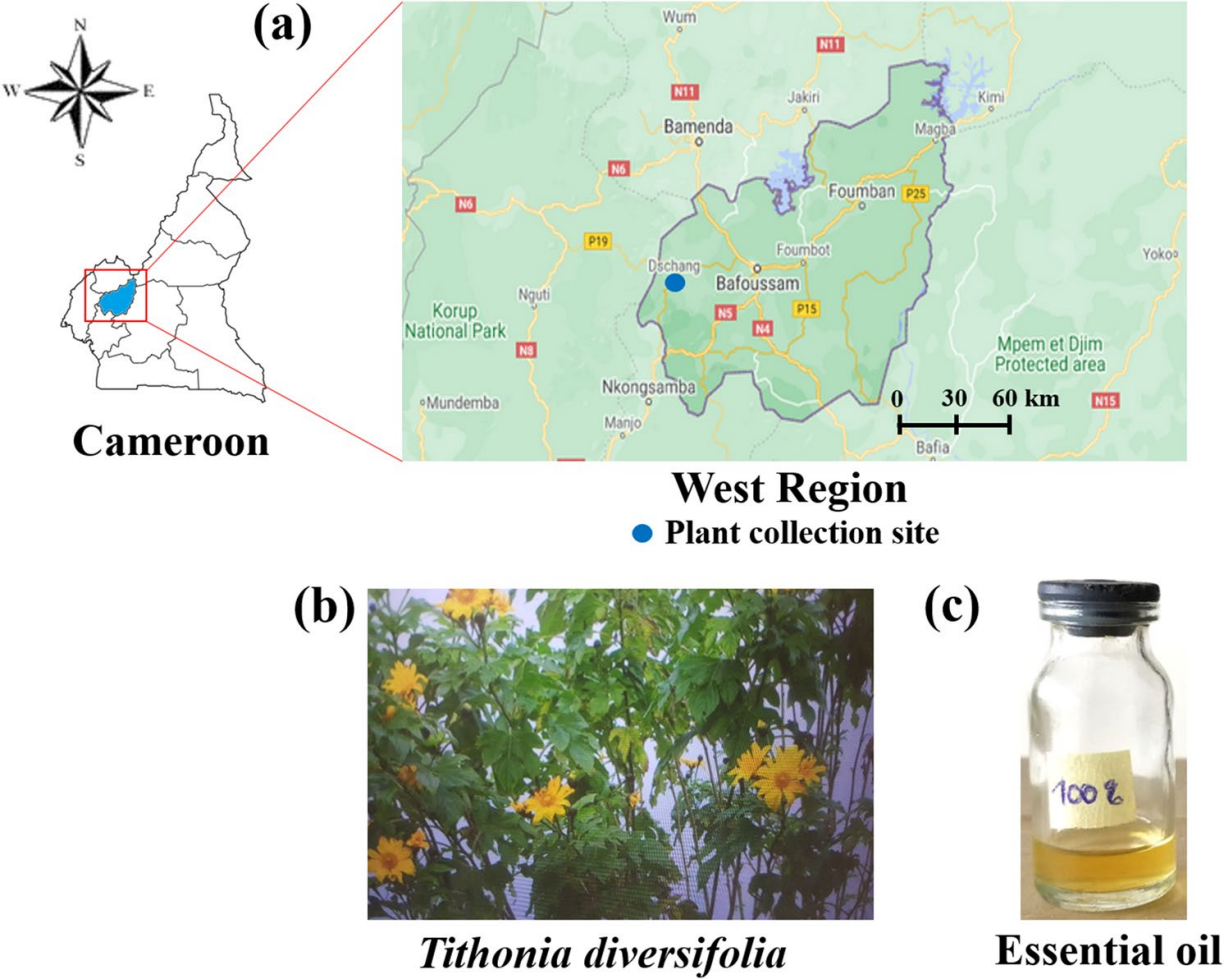
Map showing (**a**) the geographical area of collection, (**b**) *T. diversifolia* in its natural habitat, and (**c**) *T. diversifolia* essential oil-Photographs of the plants and its essential oil were provided by the authors.

The leaves of *T. diversifolia* were collected in February 2019 from natural habitat in the Fokoue Village (5°27′01.7″ latitude N, 10°05′27.5″ longitude E) located in the town of Dschang, Menoua Division, in the West Region of Cameroon (Fig. 1). Taxonomic identification of the plant was made at the National Herbarium of Cameroon in comparison with a voucher specimen (A.J.M Leeuwenberg No. 10196 registered at the National Herbarium under the No 48790/HNC). The popular use of this plant as a biopesticide and as an insect repellent against pests in plantations and, as a repellent against *A. gambiae* in the laboratory motivated the choice of this plant^24,26^.

### Extraction of the *T. diversifolia* essential oil

The essential oil was extracted in the inorganic chemistry laboratory of the Faculty of Medicine and Pharmaceutical Sciences of the University of Douala, by hydrodistillation using a Clevenger-type water steam apparatus.

After harvesting, fresh leaves (500 g) of the plant were washed with distilled water then air-dried in the dark for seven days at room temperature (29–33 °C) with daily humidity of 15.54–27.93%.. The water content (Cwat in %) was calculated using the following formula: C_wat_ = [((FVm − DPm)/FVm) × 100], where FVm is the fresh vegetable mass (g) and DPm is the dry plant mass obtained (g).

Approximately 50 kg of dried material were divided into 10 batches of 5 kg and each batch was subjected to distillation using a Clevenger-type water steam apparatus at 104 °C for three hours after the first drop of distillate appeared. Once the distillation process is completed, the distillate was left to rest for 1 h at room temperature and after this short stay in the separating funnel, two phases were formed. For each batch, 1 mL of EO on top of the water was collected with the micropipette and then introduced into a labelled bottle. The *T. diversifolia* EO are collected in 10 mL hermetically sealed glass vials wrapped with aluminum foil after dehydration of the oil-floral water mixture using anhydrous sodium sulfate (Na_2_SO_4_) then stored at + 4 °C until further analysis.

The EO yield was calculated using the following formula: C_EO_ = [(V_EO_/M_d_) × 100] ± [(ΔV_EO_/M_d_) × 100], where C_EO_ is the EO yield (mL/g), V_EO_ is the volume of EO collected (mL), ΔV_EO_ is the reading error, and M_d_ the mass of dried vegetal material (g).

### Determination of chemical composition of essential oils from *T. diversifolia*

The composition of volatile elements was determined using gas chromatography coupled with mass spectrometry (GC–MS). Separation of the EO components was performed on Hewlett-Packard silica fused capillary columns (50 cm length, 0.32 mm internal diameter, and 0.3 µm film thickness Carbowax). Helium was used as carrier gas at a flow rate of 1 mL/min. The GC column oven temperature varied from 60 to 240 °C at a rate of 7 °C/min for 20 min. Mass spectra were taken in scan mode in the range of 50–500 m/z mass to charge, operated at 70 eV, and the ion source temperature was maintained at 250 °C. The total run time was 23.82 min. The identification of compounds was done by comparing their retention index (RI) and mass spectra with those from the Wiley/National Bureau of Standards, and the National Institute of Standards and Technology libraries stored in the GC–MS database. The concentration of volatile compounds (Ci) was calculated using the following formula: %Ci = [(Ai × Fi)/Vol] where, Ai is the peak air product, Fi is the proportionality factor and Vol is the volume injected.

### Laboratory rearing of *A. coluzzii*

Eggs of *A. coluzzii* (Ngousso strain) were obtained from the “Organisation de Coordination pour la lutte contre les Endémies en Afrique Centrale, Yaounde”, Cameroon and reared in plastic bowls (300 eggs per bowl) for 24 h at the Insectarium of the Faculty of Medicine and Pharmaceutical Sciences to obtain larvae. The different larval stages obtained were fed with fish food every morning. Once the larval stages evolved into pupae, they were separated and kept in net-covered mosquito cages until emergence of adults. A cotton piece soaked in a 10% glucose solution was placed inside the cages to feed the emerged adults.

### Repellency tests

The repellent potential of the *T. diversifolia* EO was evaluated by human landing assays both in the field against wild type mosquitoes and in the laboratory using established adult female *A. coluzzii*. All volunteers freely received antimalarial prevention before experiments, as per the national guidelines for malaria management^27^.

#### Repellency activity against wild-type mosquitoes

Nightly captures of adult female mosquitoes were conducted in April 2020 and among human volunteers in the Mabanda neighbor. Mabanda is a popular neighborhood located in the third division of the city of Douala (Littoral region, Cameroon) and where insalubrity is very present and the population lives in high promiscuity. It is also a neighborhood with a large number of mosquito breeding sites due to human activities.

2 mL of EO was dripped onto each leg from the bottom of the knee to the end of the ankle then was applied by hand onto the legs of each volunteer, from the knee to the end of the ankle to cover a skin area of about 200 cm^2^ (0.01 mL/cm^2^ of EO). The nine catchers were divided into three groups of three participants. The three catchers from each group were placed one-meter away from each participant in the group while the distance between groups was variable, depending on the capture sites (next to a stream between two plank houses, next to the field and next to the hard houses inland). In each group working simultaneously in one spot, the volunteer received either *C. citratus* EO 100% known to have repellent properties (positive control), or *T. diversifolia* EO 100% (test group), or no substance (negative control) to control for any capture biases. *C. citratus* (citronella) is greatly used as repellent by populations. In contrast, DEET repellents are few used by populations due to absence of knowledge, cost and beliefs on its toxicity. In this context, we preferred using citronella-based repellents as positive control for field experiments. The capturers were instructed not to rub, touch, or wet their substance-treated legs. The field experiments were conducted between 7.00 p.m. and 11.00 p.m. (4-h exposure) with the field temperature varying from 29 to 30 °C during each capture night. The volunteers had no contact with oils, perfumed soaps, lotions or perfumes on the day of the assay. Mosquitoes species are counted and are identified using morphological traits and dichotomous keys^28–30^.

#### Mosquito rearing and repellency activity against laboratory *A. coluzzii* strains

Larvae mosquitoes were fed daily with 50 mg fish powder for 6–7 days. A piece of cotton wool soaked with glucose (30 mL, 10%) was deposited onto the net-covered for the nutrition of adults that emerged Cage. The repellency activity was assessed using the Armin cage test as described by Schreck et al., and WHO guidelines on efficacy testing of mosquito repellent for human skin^31,32^. Briefly, 3–5-day-old and adult female *A. coluzzii* mosquitoes (n = 100) without blood supply for one to two days were kept in a net-covered cage (35 cm × 35 cm × 35 cm)^24^. The DEET 30% (positive control), or vaseline (negative control), or one of the different concentrations of the *T. diversifolia* EO (10%, 30%, 50% and 100% in vaseline to reduce its volatility) were used to perform the assay as described previously^24^. Only the forearm of each volunteer was exposed and the remaining area was protected with rubber gloves. The experiments were conducted in two days between 8.00 p.m. and 03.00 a.m., and were performed during a 1-h exposure period in triplicate (three different human volunteers' hands were used per test and each volunteer received the three treatments at different times) with 1 g of each formulation puting the EO treated arm and control arms into the cages at the same time for a full hour. At the day 1, the positive control arm (DEET 30%) and the one treated with 30% EO were introduced simultaneously into the mosquito cage for each volunteer and at the day 2, the DEET was replaced by the vaseline (negative control). The exposure was stopped when the first bite of the volunteer was noted. Before the experiment, the volunteer's forearm was treated with petroleum jelly as control and exposed for 30 s to check for repulsion. If at least two mosquitoes landed on the arm, the repellency assay is continued as this means that petroleum jelly has no repellent effect on mosquitoes, so it would not affect the repellent activity of the EO of *T. diversifolia* to be tested. Likewise, the volunteers had no contact with other substances (i.e., body oils and lotions, perfumed soaps, fragrances) on the day of the assay.

#### Repellency assay outcomes

Field and laboratory-based repellent activities were assessed by determining the repulsion rate and the protection time for each substance. The repulsion rate (Re) was determined using the following formula: Re = [(N_C_ – N_EO_)/N_C_] × 100, where N_C_ is the number of mosquitoes captured by volunteers treated with negative control, and N_EO_ is the number of mosquitoes captured by volunteers treated with the test substance. This rate was used to assess reduction in the attractiveness of mosquitoes to humans treated with the substance tested. The protection time is the time interval (min) between the application of the substance and the first mosquito bite.

### Statistical analysis

Data were keyed in an Excel spreadsheet (Microsoft Office, USA) and then exported to the statistical package for social sciences v16 (SPSS, IBM, Inc., IL, Chicago, USA) and GraphPad v5.03 (GraphPad Prism, Inc., San Diego, California, USA) software for statistical analysis. Non parametric Mann–Whitney and Kruskal–Wallis tests were used to compare mean values between groups while Fisher’s exact and Pearson’s independence chi-square were used to compare proportions. Level of statistical significance was set at p < 0.05.

### Ethics approval and consent to participate

The study was conducted in accordance with ethical guidelines related to research on humans in Cameroon. The study received ethical clearance from the Institutional Committee of Ethics for Research for Human Health of the University of Douala (no. 1976/CEI-UDo/06/2019/T). Before enrollment, subjects were informed on the purpose and process of the investigation (background, goals, methodology, study constraints, data confidentiality, and rights to opt out from the study), and signed informed consent was obtained from all those who agreed to participate in the study in accordance with the Helsinki Declaration. Participation was voluntary, anonymous and without compensation.

Collection of wild plant material was carried out in accordance with national guidelines and Cameroonian legislation (provisions of the law on forest and environmental management in Cameroon No 94/01 of 20 January 1994 relating to the forest, fauna and fishing regime in Cameroon and explicitly recognizing the rights of local populations use on various forest products) and an identification certificate was obtained at the National Herbarium of Cameroon.

## Results

### Phytochemical screening of the *T. diversifolia* essential oil

The moisture content of the leaves of *T. diversifolia* was 81% and the yellow essential oil yield was 0.02% after hydrodistillation of 50 kg of dry leaves of *T. diversifolia*.

The GC–MS analysis revealed the presence of 29 compounds in the *T. diversifolia* EO (yellow pale-colored), with d-limonene (20.06%), α-Copaene (10.29%) and o-Cymene (10.0%) as the most represented (Table 1). The 29 compounds identified belong to four groups namely monoterpenes, sesquiterpenes, phenylbutane derivatives and triterpenes, which accounted for 54.13%, 35.22%, 8.87% and 1.78% of the compounds, respectively.

**Table 1:** Chemical composition of the *T. diversifolia* essential oil.

No	RI (min)	Compounds	%
1	13.02	Bicyclo [7.2.0] undec-4-ene, 4,11,11-trimethyl-8-methylene-, [1R-(1R*, 4Z,9S*)]	0.68
2	13.16	Modephene	2.28
3	13.28	(1R, 3aS,5aS,8aR)-1,3a,4,5a-Tetramethyl-1,2,3,3a,5a,6,7,8-octahydrocyclopenta[c]pentalene	0.30
4	13.63	α-Copaene	10.29
5	13.87	Caryophyllene	8.44
6	15.63	(E)-2-((8R,8aS)-8-8aDimethyl-3,4,6,7,8,8a-hexahydronaphtalen-2-1H)-ylidene) propan-1-ol	1.07
7	16.22	Isocaryophilene	8.73
8	16.64	1a,2,6,7,7a,7b-hexahydro-1,1,7,7a-tetramethyl-, [1aE-(1aà,7à, 7aà,7ba]-1H-cycloprapa[a]naphthalene	1.58
9	16.76	o-Cymene	10.00
10	17.89	α-Guaiene	2.14
11	4.67	α-Pinene	7.72
12	6.29	d-Limonen	20.06
13	6.82	(+)-3-Caren	0.91
14	7.56	Naphtalen	4.01
15	8.53	1,3,8-p-Menthetriene	0.85
16	9.42	α-Terpineol	1.86
17	10.03	2,6,6-trimethyl-1-Cyclohexane-1-carboxaldehyde	0.86
18	10.90	Cis-p-Mentha-2,8-dien-1-ol	0.91
19	14.47	Oxide-1-aromadendrene	1.90
20	14.93	n-Pentadecanol	8.77
21	5.45	6-Methyl-5-Hepten-2-one	2.76
22	7.63	Nonanal	0.31
23	8.71	2,6-Dimethyl-3,5-Heptadien-2-ol	0.83
24	9.17	4-Terpinenyl acetate	0.10
25	14.11	4-(2,6,6-Trimethyl-1-cyclohewen-1-yl)-2-Butanone	0.61
26	20.57	6,10,14-Trimethyl-2-Pentadecanone	4.26
27	25.30	2-Methyl-eicosane	0.86
28	21.67	3-Methyl-2-(3,7,11-trimethyldodecyl) furane	0.88
29	24.25	3,7,11,15-Tetramethyl-2-hexadecen-1-ol	0.90

The table represents information on the chemical composition of the *T. diversifolia* essential oil. RI: retention index; A number was used to differentiate between Sesquiterpernes (1 to 10), Monoterpenes (11 to 20), Phenylbutane derivatives (21 to 27) and the Diterpenes (28 and 29). Percentage of each compound is presented in the last column.

### Field repellent activity

**Figure 2:**
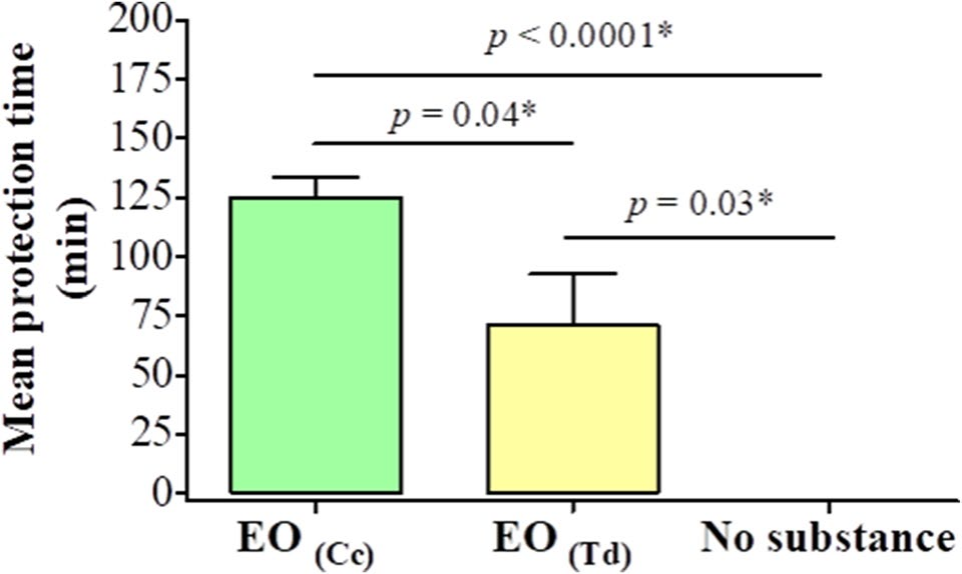
Mean protection time on field of the different substances tested. EO_(Cc)_: Essential oil of *C. citratus* (positive control); EO_(Td)_: Essential oil of *T. diversifolia;* Mann–Whitney test was used to make pairwise comparisons; *Statistically significant at p < 0.05.

The mean time of protection against mosquito bites was significantly higher in volunteers treated with the *C. citratus* EO as compared to those treated with the *T. diversifolia* EO (125 min versus 71 min, p = 0.04) (Fig. 2).

**Figure 3:**
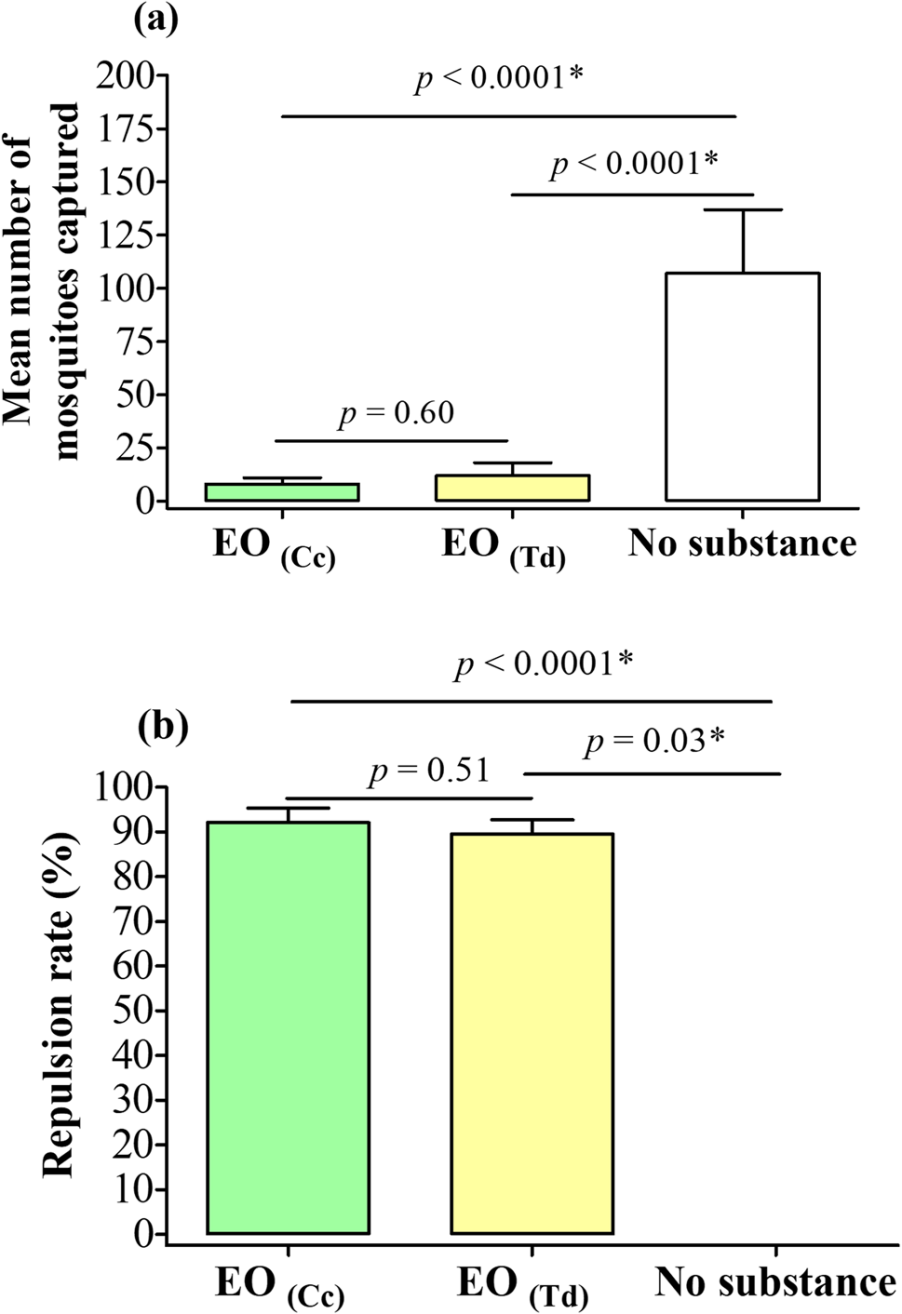
Mean number of mosquitoes captured (**a**), and mean repulsion rate (**b**) in the different groups evaluated. EO _(Cc)_: Essential oil of *C. citratus* (positive control); EO _(Td)_: Essential oil of *T. diversifolia*; Pearson’s independence chi-square test was used to compare the groups; *statistically significant at p < 0.05.

The mean number of mosquitoes captured were similar between capturers treated with the *C. citratus* EO or *T. diversifolia* EO (p = 0.60) (Fig. 3a). Likewise, repulsion rate between the two groups were statistically similar (92.1% for *C. citratus*–treated group, and 89.5% for *T. diversifolia*–treated group, p = 0.51) (Fig. 3b).

**Figure 4:**
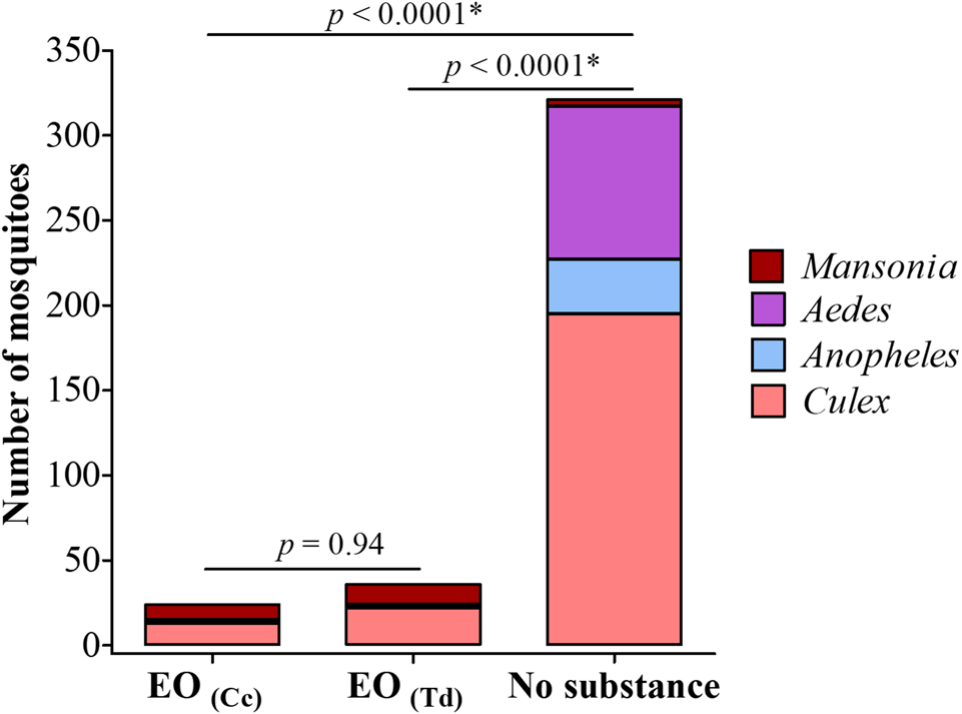
Entomofauna captured in the different groups evaluated. EO_(Cc)_: Essential oil of *C. citratus* (positive control); EO_(Td)_: Essential oil of *T. diversifolia*; Pearson’s independence chi-square test was used to compare the groups; *statistically significant at p < 0.05.

The analysis of the entomofauna captured revealed a similar distribution of *Culex*, *Anopheles* and *Mansonia* mosquitoes between the two groups treated with the EO (Fig. 4). Regarding *Anopheles* mosquitoes collected in the groups, these were predominantly identified as *A. gambiae s.l*.

It should be noted that minor burning sensations were reported only in volunteers treated with the *C. citratus* EO. No adverse signs/symptoms were observed in those treated with the *T. diversifolia EO*.

### In vitro repellent activity

**Figure 5:**
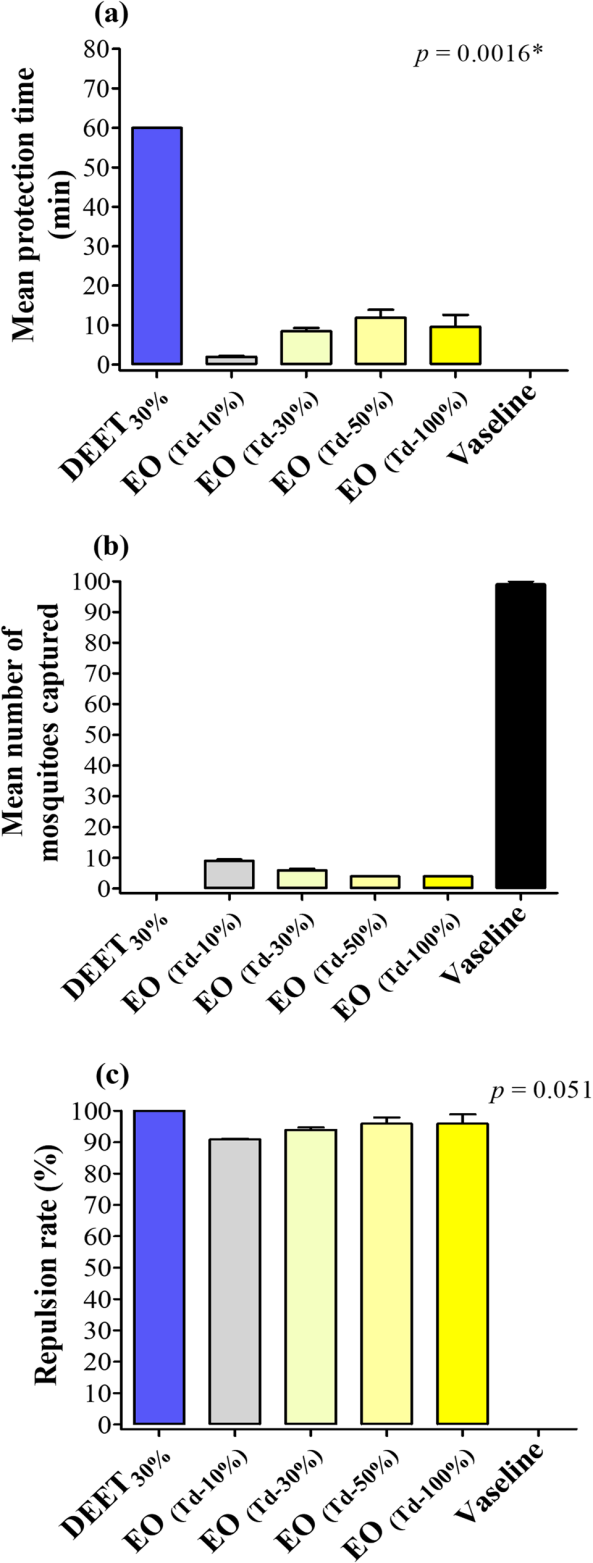
Findings from the in vitro repellency activity. (**a**) Mean protection time, (**b**) Number of mosquitoes captured after 1 h, (**c**) Repulsion rate after 1 h. DEET: N, N-diethyl-3-methylbenzamide (positive control); EO _(Td-X%)_: Essential oil of *T. diversifolia* at dose 10%, 30%, 50% and 100%; Kruskal–Wallis test was used to make comparisons between the groups; *Statistically significant at p < 0.05.

Finding from the in vitro repellency assay showed that the time of protection from biting was on average higher in the group of capturers treated with DEET as compared to the other groups treated with varying concentration of the *T. diversifolia* EO, and the difference was statistically significant (p = 0.0016) (Fig. 5a). In contrast, no difference was found in terms of the number of mosquitoes captured and repulsion rate between DEET-treated group and the *T. diversifolia–*treated groups (p > 0.05) (Fig. 5b,c). No allergic events were reported during the laboratory experiments.

## Discussion

The emergence of mosquito populations resistant to synthetic insecticides and commercially available repellents hinders their use at the population level for control of mosquito-borne diseases such malaria. Unlike their synthetic counterparts, there is no evidence on emergence of resistance to natural substances. In this context, there is a need for new molecules with good repellent potentials and safety. We therefore evaluated the repellent potential of EO from leave parts of *T. diversifolia*, a plant traditionally used in Cameroon for treating chickenpox, and in other countries (e.g., Mexico and Nigeria) for treating malaria and other diseases^32–35^.

The yield of *T. diversifolia* EO (0.02%) differs from that found for the plant collected in another city in Cameroon in a previous study^36^. This finding is consistent with other previous studies showing a difference in yields depending on plant's harvest location, harvest period, leaf condition, drying or extraction time or technique with a yield that varies between 0.01% and 0.1% for essential oils extracted from leaves^24,37–39^. Another study revealed that in addition to the storage time, the transport conditions also impact the EO yield of this plant^40^.

Hydrogenated monoterpenes and sesquiterpenes were the main compounds found in the *T. diversifolia* EO. This finding is also consistent with previous reports on the chemical composition of the plant EO from other regions of Cameroon and beyond^24,33,35,38,41^, though other previous reports from Cameroon outlined a predominance of sesquiterpenes in flower EO^36^. Our detailed analysis of the chemical composition identified 3 of the 29 compounds at high levels viz. (d)-α-limonene (monoterpenes), α-Copaene (sesquiterpenes) and o-Cymene (sesquiterpenes). Lamaty and colleagues found that (Z)-β-ocimene (40.2%) was the main compound from EO of *T. diversifolia* collected in the town of Yaoundé, Centre region of Cameroon^36^. This discrepancy highlights the role of environment in shaping biological development of plants, and thus its impact on their chemical composition and biological activities^24,37,41^.

Our study is the first to evaluate the repellent activity of *T. diversifolia* against *A. coluzzii*, a major malaria vector in Africa. We demonstrated the repellent potential of this plant against laboratory strains of *A. coluzzii* and against natural mosquito bites in field studies. Our findings support previous studies on the same plant that reported repellent activities of its EO fractions against *A. gambiae*, *Aedes aegypti* and *Culex quinquefasciatus*^23^. This repellent activity in natural condition was higher than that observed against the laboratory strain, probably due to the time between the harvest, the time of extraction (4 months after harvest) of essential oil, the natural condition test (15 days after extraction) and the laboratory test (1 month after the natural conditions test) that would have an impact on the volatile properties of certain terpenic compounds^38^. The high content of *C. citratus* EO in α-pinene (34.4% vs 7.72% for *T. diversifolia* EO) could explain it strong repellent activity (92% vs 89.5%) compared to the *T. diversifolia* EO rich in (d)-α-limonene, and studies have shown that α-pinene is a powerful repellent^42–44^. The difference in the duration between the harvest period and the extraction could have induced the volatility of compounds (difference in concentration) and the conformation change of certain compounds of the *T. diversifolia EO* tested in our study, as has been suggested by Walker et al.^41^  The latter could also explain the low repellent activity of *T. diversifolia* EO compared to *C. citratus* EO. Besides, it would also be interesting to conduct further studies to identify the chemical compounds responsible for repellent activity of *T. diversifolia* along with elucidating mechanism of action. Given high content of (d)-α-limonene, this compound is probably greatly involved in the repellent activity of *T. diversifolia* EO, possibly in association with other major compounds found (α-Copaene and o-Cymene).

The results showed a shorter protection time in *T. diversifolia* EO-treated volunteers compared to the individuals treated with the positive control for both the laboratory (DEET) and field (*C. citratus* EO) assays. In natural conditions, the mean protection time was 71 min for *T. diversifolia* EO and 125 min for the positive control. These findings suggest that the *T. diversifolia* EO has similar repellency, but that the volatile nature of its compounds greatly impacted the protection time of *T. diversifolia* EO. These preliminary results on protection time against wild mosquitoes suggest that *T. diversifolia* EO should be used each hour in to maintain its repellent activity, which is not achievable in practice. In this context, it would be helpful to develop release-control formulations in order to reduce its the volatility.

In the laboratory, the concentrations of the *T. diversifolia* EO at 50% provided a better time protection (11.9 min) against *A. coluzzii* than the 100% extract (9.6 min), but this time was significantly less than that of DEET at 30%. Oyewole et al. in Nigeria^24^ had found protection times of 120, 160 and 210 min, respectively for the 10%, 50% and 100% formulations, higher than those found in our study. These results corroborate with previous study suggesting the influence of the solvent used in the formulations; unlike hexane which was used by Oyewole et al., the petroleum jelly used in our study would slow the volatility of the EO^24^. Variability in the sensitivity of an EO against *Anopheles* species has also been reported, *A. gambiae* was more sensitive to repellents than *A. coluzzii*^18^. One solution to improve protection time of *T. diversifolia* EO could be to develop controlled-release formulations in order to increase the duration of repellence activity. The volatile nature of the compounds of the EO as well as a combination of other factors such as chemical composition, natural resistance of the mosquito vector species and experimental conditions can explain discrepancies observed between the groups treated and positive control.

## Conclusion

The present preliminary study showed promising repellent activity of the leave *T. diversifolia* EO against *A. coluzzii* and, suggests that it could be used as an effective vector control tool at the individual level to complement conventional control methods at the community level. However, the limited time of protection compared to controls outlines the need for extensive research on development of release-control formulations and inocuity before its potential introduction as a commercial repellent.

## Data Availability

All data underlying the findings and the conclusions are included within the manuscript.

## Abbreviations

*A.*: *Anopheles*
ACT: Artemisinin-based combination therapy
DEET: N,N-Diethyl-meta-toluamide
EO: Essential oil
EO: Essential oil
FMPS: Faculty of Medicine and Pharmaceutical Sciences
GC–MS: Gas chromatography–mass spectrometry
LLIN: Long-lasting insecticide-treated net
RI: Retention index
WHO: World Health Organization
